# *Escherichia coli* of sequence type 3835 carrying *bla*_NDM-1_, *bla*_CTX-M-15_, *bla*_CMY-42_ and *bla*_SHV-12_

**DOI:** 10.1038/srep12275

**Published:** 2015-07-21

**Authors:** Yu Feng, Ping Yang, Yi Xie, Xiaohui Wang, Alan McNally, Zhiyong Zong

**Affiliations:** 1 Center of Infectious Diseases, West China Hospital, Sichuan University, Chengdu, China; 2 Division of Infectious Diseases, State Key Laboratory of Biotherapy, Chengdu, China; 3 Laboratory of Clinical Microbiology, Department of Laboratory Medicine, West China Hospital, Sichuan University, Chengdu, China; 4 Department of Infection Control, West China Hospital, Sichuan University, Chengdu, China; 5 Pathogen Research Group, Nottingham Trent University, Nottingham, UK

## Abstract

New Delhi metallo-β-lactamase (NDM) represents a serious challenge for treatment and public health. A carbapenem-resistant *Escherichia coli* clinical strain WCHEC13-8 was subjected to antimicrobial susceptibility tests, whole genome sequencing and conjugation experiments. It was resistant to imipenem (MIC, >256 μg/ml) and meropenem (MIC, 128 μg/ml) and belonged to ST3835. *bla*_NDM-1_ was the only carbapenemase gene detected. Strain WCHEC13-8 also had a plasmid-borne AmpC gene (*bla*_CMY-42_) and two extended-spectrum β-lactamase genes (*bla*_CTX-M-15_ and *bla*_SHV-12_). *bla*_NDM-1_ and *bla*_SHV-12_ were carried by a 54-kb IncX3 self-transmissible plasmid, which is identical to plasmid pNDM-HF727 from *Enterobacter cloacae*. *bla*_CMY-42_ was carried by a 64-kb IncI1 plasmid and *bla*_CTX-M-15_ was located on a 141-kb plasmid with multiple F replicons (replicon type: F36:A4:B1). *bla*_CMY-42_ was in a complicated context and the mobilisation of *bla*_CMY-42_ was due to the transposition of IS*Ecp1* by misidentifying its right-end boundary. Genetic context of *bla*_NDM-1_ in strain WCHEC13-8 was closely related to those on IncX3 plasmids in various *Enterobacteriaceae* species in China. In conclusion, a multidrug-resistant ST3835 *E. coli* clinical strain carrying *bla*_NDM-1_, *bla*_CTX-M-15_, *bla*_CMY-42_ and *bla*_SHV-12_ was identified. IncX3 plasmids may be making a significant contribution to the dissemination of *bla*_NDM_ among *Enterobacteriaceae* in China.

Carbapenems have long served as reliable and potent agents against Gram-negative bacilli. However, a variety of enzymes produced by bacteria are able to hydrolyse carbapenems, termed carbapenemases. New Delhi metallo-β-lactamase (NDM) is a type of carbapenemase and confers resistance to all β-lactams except monobactams^1^. NDM was first found in a *Klebsiella pneumoniae* isolate in 2008^2^ and since then bacterial isolates producing NDM have been identified worldwide, representing a serious challenge for treatment, infection control and public health. In addition to India, China is a common place from which NDM-producing isolates are identified. In China, NDM was initially seen in *Acinetobacter* spp^3^. but NDM-producing *Enterobacteriaceae* have been increasingly reported^4^. Among carbapenem-resistant *Enterobacteriaceae* in China, *K. pneumoniae* is the most common species with 10% of 12,121 *K. pneumoniae* clinical isolates collected from 16 major teaching hospitals in 2013 resistant to imipenem^5^. In contrast, carbapenem resistance is relatively uncommon in *Escherichia coli* in China and only 1% of 16,794 *E. coli* clinical isolates from the 16 hospitals in 2013 were resistant to imipenem^5^. During routine clinical duties, a carbapenem-resistant *E. coli* clinical isolate was encountered. This strain was then characterised and is reported here.

## Results and Discussion

Clinical strain WCHEC13-8 was recovered on November 2012. This strain was identified as *E. coli* and was resistant to imipenem (Minimum inhibitory concentration [MICs], >256 μg/ml), meropenem (MIC, 128 μg/ml), ceftazidime (MIC, >256 μg/ml), ciprofloxacin (MIC, 256 μg/ml) but was intermediate to amikacin (MIC, 32 μg/ml) determined by the microdilution method. It was also resistant to ampicillin-sulbactam, aztreonam, cefazolin, cefepime, cefotaxime, cefoxitin, ceftriaxone, gentamicin, piperacillin-tazobactam, tobramycin and trimethoprim-sulphamethoxazole determined by Vitek II. *bla*_NDM_ was the only carbapenemase-encoding gene that was detected and sequencing the complete coding sequence of the gene revealed that it was *bla*_NDM-1_.

Strain WCHEC13-8 was subjected to whole genome sequencing on the Illumina HiSeq platform. A total of 7,149,960 reads and 643,496,400 bases were obtained from the genome sequencing with a 49.8% GC content. Reads were assembled to 268 contigs that were ≥500 bp in length (N50 metric, 118,912 bp) and contained 5,145,308 bp nucleotides. To identify whether strain WCHEC13-8 had any new genes or not, its genome sequence was compared to other *E. coli* genome sequences including 83 complete and 2,159 draft genomes available in GenBank. Despite comparing with such a large number of *E. coli* genomes, the strain still had three unique genes that were located on contigs belonging to the chromosome. Two of the three genes were clustered together and resided in the centre of a 149-kb large contig, which was part of the chromosome as it shows significant identity (74% coverage and 97% identity) with the chromosome of *E. coli* strain E2348/69 (GenBank accession number FM18568) identified using the BLAST program. The two novel genes encoded hypothetical proteins with 420 and 346 amino acids, respectively. Unfortunately, the function of the two hypothetical proteins were not able to be predicted using both Protein BLAST and InterProScan programs. The remaining unique gene was 2,976 bp on a small 3,322 bp contig and contained both an endonuclease/exonuclease/phosphatase and a reverse transcriptase domain identified using the InterProScan program.

Strain WCHEC13-8 belonged to the phylogenetic group A and a new Sequence type (ST), ST3835 (*adk*-*fumC*-*gyrB*-*icd*-*mdh*-*purA*-*recA* allele numbers, 10-4-4-411-8-13-73). Multiple ST3835 isolates have been found in Korea in 2013, which is later than strain WCHEC13-8 (http://mlst.warwick.ac.uk/mlst/dbs/Ecoli). ST3835 has a single allele different from ST1284 (10-4-4-8-8-13-73) and ST3835 *E. coli* has been recovered from a human in Spain and from a dog in Germany (http://mlst.warwick.ac.uk/mlst/dbs/Ecoli). ST3835 belongs to the ST10 complex. Besides ST3835, STs belonging to the ST10 complex (ST40, ST167, ST205, ST744 and ST1237), ST88 complex (ST224 and ST410), ST101 complex (ST101) or ST361 complex (ST361) have been found to carry *bla*_NDM-1_ in China^4^,^6^,^7^,^8^,^9^. The diversity of clonal background of *E. coli* carrying *bla*_NDM-1_ suggests that the dissemination of *bla*_NDM-1_ in China was unlikely to be mainly mediated by a particular strain.

In addition to *bla*_NDM-1_, strain WCHEC13-8 had other resistance genes including *bla*_CTX-M-15_ (an extended-spectrum β-lactamase [ESBL] which is globally distributed), *bla*_SHV-12_ (an ESBL gene), *bla*_CMY-42_ (a plasmid-borne AmpC cephalosporinase gene), *bla*_OXA-1_ (a non-ESBL oxacillinase gene), *bla*_ampC_ (a chromosome-based AmpC gene), *aac(6′)-Ib*-cr (encoding an aminoglycoside acetyltransferase with low-level activity against fluoroquinolones), *aac(3)-II* (encoding an aminoglycoside acetyltransferase), *mph* (encoding macrolide 2′-phosphotransferase I) and *tetB* (conferring resistance to tetracycline). Of note, CMY-42, the AmpC enzyme encoded by *bla*_CMY-42_, differs from CMY-2 by a single amino acid substitution, i.e. Ser for Val at Ambler’s position 211^10^. The co-existence of *bla*_NDM-1_, *bla*_CMY-42_, *bla*_CTX-M-15_ and *bla*_SHV-12_, all of which encode a β-lactamase with the ability to hydrolyse broad-spectrum cephalosporins, in a single isolate has not been reported before. The co-presence of *bla*_NDM-1_, *bla*_CMY-42_ and *bla*_CTX-M-15_ has been found in an *E. coli* of ST101 from a two-year-old child who had recently travelled to India^11^, while two *E. coli* isolates of ST167 in China have been found carrying *bla*_NDM-1_, *bla*_CMY-42_ and *bla*_CTX-M-14_^6^. In addition, *bla*_CMY-42_ has also been found in two *E. coli* isolates from a river in India (GenBank accession numbers KJ661335 and KJ661336) and one from a surgical wound at a university hospital in Germany^10^, but STs of the three *E. coli* isolates have not been reported. Of note, a report from Egypt also claimed that *bla*_CMY-42_ has been detected in 8 *E. coli* isolates^12^, but the gene detected was 99% similar to *bla*_CMY-42_ and the amino acid sequence of CMY was not available for analysis. Therefore, It remains unclear whether these Egyptian isolates carried *bla*_CMY-42_ or another variant.

Strain WCHEC13-8 had three plasmids, which were approximately 50, 60 and 140 kb in size, as revealed by S1 nuclease pulse-field gel electrophoresis (S1-PFGE). The three plasmids were completely circularised using PCR and Sanger sequencing. *bla*_NDM-1_ and *bla*_SHV-12_ were carried by a 54-kb plasmid, assigned pNDM1_EC8 here, of the IncX3 group. *bla*_CMY-42_ was located on a 64-kb IncI1 plasmid, assigned pCMY42_EC8 here, while *bla*_CTX-M-15_ was carried by a 141-kb IncF plasmid, assigned pCTXM15_EC8.

pCTXM15_EC8 contained multiple F replicons including one FIA, one FIB and two FII replicons. Based on the replicon sequence typing (RST) for IncF plasmids^13^, the replicon types of pCTXM15_EC8 were F36:A4:B1 (FII 36, FIA 4 and FIB 1) with both FII replicons belonging to the same type. The F36:A4:B1 type has also been seen in plasmid p6409-151.583 kb (GenBank accession number CP010372), which was recovered from an *E. coli* strain in Colombia. However, p6409-151.583 kb has an additional F31 type FII replicon and does not carry *bla*_CTX-M_. Furthermore, the link between pCTXM15_EC8 and p6409-151.583 kb could not be established and the origin of pCTXM15_EC8 remains unknown.

pCMY42_EC8 harboured four of the five loci used for the IncI1 plasmid MLST scheme^14^. The four loci and their allele numbers were *repI1* (IncI1 replicon) 4, *ardA* (alleviation of restriction of DNA) 5, *trbA* (a gene essential for conjugative transfer) 15 and *pilL* (a pili gene) 3. The *sogS* (suppressor of *dnaG* mutation) locus was absent from pCMY42_EC8 and therefore no ST could be assigned. Nonetheless, pCMY42_EC8 was closely related to ST55 (*repI1*-*ardA*-*trbA*-*sogS-pilL* alleles 4-5-15-11-3) and ST141 (4-5-15-4-3). Both STs comprise IncI1 plasmids carrying *bla*_CMY-42_ found in Taiwan (pubmlst.org/plasmid).

**Figure 1:**
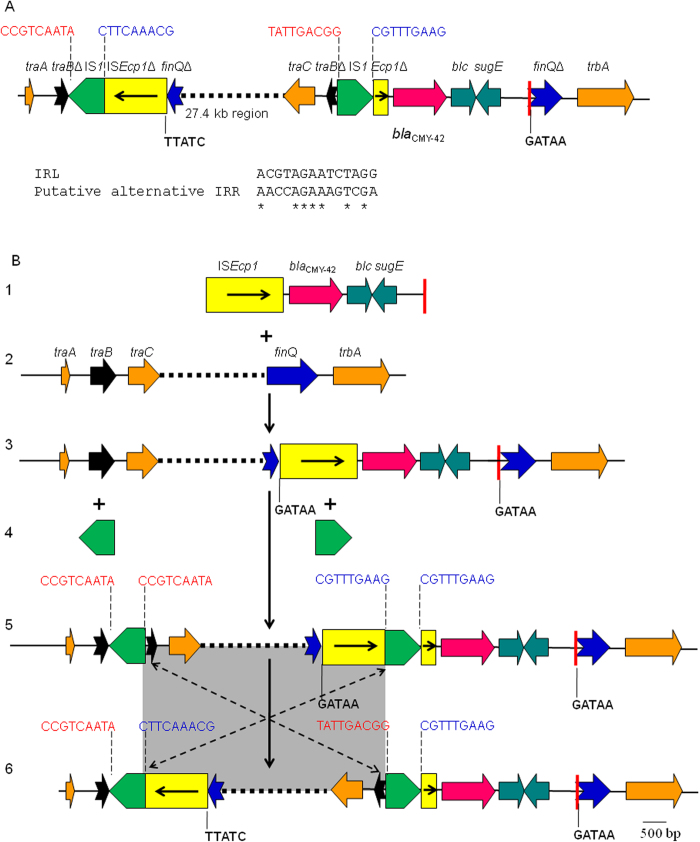
Genetic context of *bla*_CMY-42_ and its formation on pCMY42_EC8. Panel A, The context of *bla*_CMY-42_. *Δ* and shapes with a forked tail represent truncated genes or insertion sequences. The broken line represents a 27.4 kb region between *traC* and *finQ*, which is not scaled. The putative alternative IRR (IRR2) of IS*Ecp1* is depicted as a red pole and the alignment of the IRL of IS*Ecp1* and the IRR2 is shown. IS*Ecp1* and *traB* are both interrupted into two parts. The 9-bp nucleotide repeats belonging to IS*Ecp1* are depicted in blue and those of *traB* are in red, while the 5-bp nucleotide repeats belonging to *finQ* is shown in bold. Panel B, The proposed scheme for the formation of the context of *bla*_CMY-42_. The IS*Ecp1*-*bla*_CMY-42_-IRR2 region (structure 1) is inserted into the *finQ* gene of the *traA*-*trbA* region (structure 2) on an IncI1 plasmid and then generates the structure 3 with the 5-bp characteristic DR (GATAA). Two copies of IS*1* (structure 4) are then inserted into *traB* and IS*Ecp1*, respectively, generating the structure 5 with the 9-bp DR (CCGTCAATA for *traB* and CGTTTGAAG for IS*Ecp1*), which is the characteristic of IS*1* insertion. The subsequent homologous recombination between the two copies of IS*1* mediates the inversion (shown by two broken arrows) of the intervening region (grey part) and then forms the context of *bla*_CMY-42_ on pCMY42_EC8 (structure 6).

The genetic context of *bla*_CMY-42_ has not been described before. On pCMY42_EC8, *bla*_CMY-42_ is adjacent to the insertion sequence IS*Ecp1*, which was truncated by the insertion of IS*1*, at upstream and a gene encoding outer membrane lipoprotein at downstream (Panel A, Fig. 1). The remaining part of IS*Ecp1* was found 24.7-kb further upstream of *bla*_CMY-42_ and was also truncated by IS*1*. After carefully examining the flanking sequences of IS*Ecp1* and IS*1*, it became evident that the complex genetic context of *bla*_CMY-42_ has been formed by the transposition of IS*Ecp1* and the insertion of two copies of IS*1* followed by homologous recombination between the two IS*1* (Panel B, Fig. 1) as explained as below. First, both of the two IS*1* were flanked by a remnant of IS*Ecp1* and a part of the *traB* gene, which is involved in plasmid conjugation. The two remnants of IS*Ecp1* form a complete IS*Ecp1* plus a 9-bp repeat, which abuts the two IS*1* and is characteristic of the insertion of IS*1*. Similarly, the two parts of *traB* make the complete *traB* plus the characteristic 9-bp repeat. It is therefore clear that both IS*Ecp1* and *traB* had been interrupted by the insertion of IS*1* and the subsequent homologous recombination between the two copies of IS*1* could result in the inversion of the intervening region (Panel B, Fig. 1). Second, it has been proven that a single copy of IS*Ecp1* is able to mobilise its downstream genetic components by using alternative sequences as the right-hand inverted repeat (IRR)^15^. By comparing genetic contexts of *bla*_CMY-2_ and its variants available in GenBank using BLAST, the same 4 kb element comprising IS*Ecp1*, *bla*_CMY_, *blc* (encoding an outermembrane lipoprotein) and *sugE* (encoding a quaternary ammonium compound-resistance protein) has been found on different plasmids but with varied abutting sequences. This element is bounded by a 14 bp sequence (AACCAGAAAGTCGA) at one end, which shows some similarity with the left-hand inverted repeat (IRL) of IS*Ecp1* (Panel B, Fig. 1) and might have served as the alternative IRR (IRR2) for IS*Ecp1* to mobilise *bla*_CMY_ to different locations. Indeed, when we put the two remnants of IS*Ecp1* back together, we found that the *finQ* gene (the transcriptional inhibitor of plasmid transfer) has been interrupted by the IS*Ecp1*-*bla*_CMY-42_-IRR2 region with the presence of 5-bp direct target repeats (DR), which characterises the insertion of IS*Ecp1* (Panel B, Fig. 1). This confirms that the mobilisation of *bla*_CMY-42_ has been mediated by IS*Ecp1* by misidentifying its IRR.

In strain WCHEC13-8, *bla*_NDM-1_ was able to be transferred to *E. coli* strain J53, suggesting that pNDM1_EC8 was a self-transmissible plasmid. None of the other resistance genes listed above except *bla*_SHV-12_ were co-transferred with *bla*_NDM-1_. PCR-based replicon typing (PBRT) failed to assign pNDM1_EC8 to a replicon type as all replicon typing PCR reactions were negative. pNDM1_EC8 was an IncX3 plasmid and IncX3 has not been integrated into the PBRT scheme. pNDM1_EC8 is identical to pNDM-HF727 (GenBank accession number KF976405), an IncX3 plasmid carrying *bla*_NDM-1_ from an *Enterobacter cloacae* strain in China^9^.

**Figure 2:**
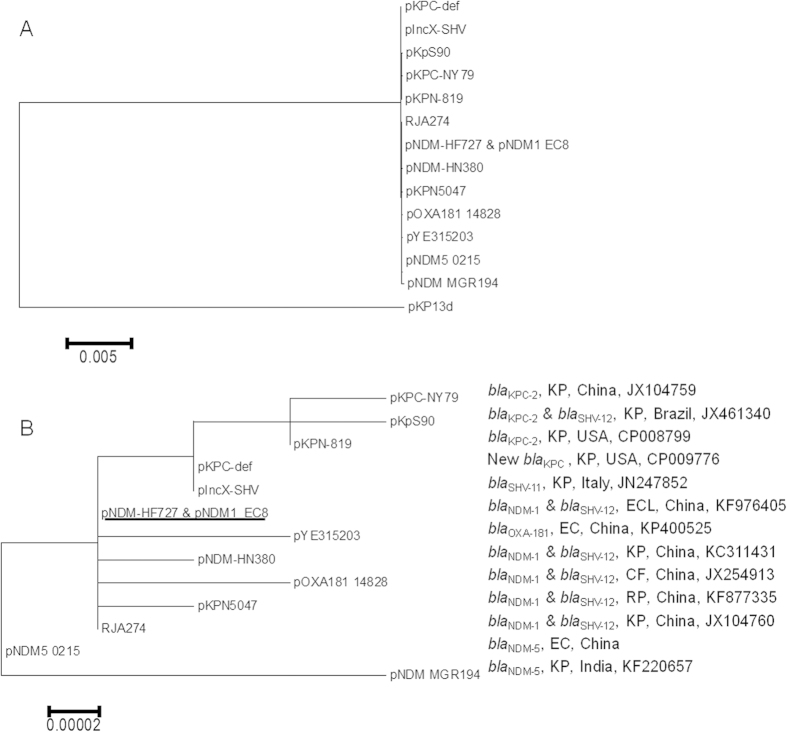
Phylogenetic trees of IncX3 plasmids. The trees are generated using MEGA based on the complete sequence of IncX3 plasmids. Panel A, a tree of IncX3 plasmids including pKP13d, which carries *bla*_KPC-2_ in a *K. penumoniae* strain from Brazil (GenBank accession number CP003997). pKP13d is distinct from other IncX3 plasmids in sequence. Panel B, a higher resolution tree of IncX3 plasmids excluding pKP13d. The carbapenemase and ESBL genes, the host species, the locations of recovery and the GenBank accession number of each plasmid are shown. For host species, CF refers to *Citrobacter freundii*, EC to *E. coli*, ECL to *E. cloacae*, KP to *K. pneumoniae* and RP to *Raoultella planticola*. The plasmid carrying *bla*_NDM-1_ identified in this study is identical to pNDM-HF727, which is underlined. The GenBank accession number of pNDM5_0215^17^ is not available.

IncX3 plasmids are narrow-host-range plasmids of *Enterobacteriaceae*^16^. A few IncX3 plasmids carrying ESBL genes (*bla*_SHV-11_ or *bla*_SHV-12_) and/or carbapenemase genes (*bla*_KPC-2_, *bla*_NDM-1_ or *bla*_NDM-5_) from various species of *Enterobacteriaceae* in several countries have been completely sequenced^9^,^17^,^18^,^19^,^20^,^21^. In particular, IncX3 plasmids carrying *bla*_NDM-1_ and *bla*_SHV-12_ have been identified in various species of the *Enterobacteriaceae* at multiple locations in China. The completely-sequenced IncX3 plasmids except pKP13d are almost identical in their backbone sequences with less than 9 single nucleotide variations between any two plasmids (Fig. 2). The remarkable similarity between IncX3 plasmids suggests that a common IncX3 plasmid has spread to different continents, acquired the carbapenemase gene that is prevalent locally, e.g. *bla*_KPC-2_ in USA and *bla*_NDM-1_ in China, and then were transformed as a vehicle mediating the interspecies spread of carbapenemase genes in local settings.

IncX3 plasmids carrying *bla*_NDM-1_ and *bla*_SHV-12_ have also been found in strains causing an outbreak in China^22^. IncX3 plasmids carrying *bla*_NDM-1_ and *bla*_SHV-12_ have also been found among the *Enterobacteriaceae* in the United Arab Emirates (UAE). Of note, one of the UAE strain was recovered two years earlier than the first Chinese strain that harboured an IncX3 plasmid carrying *bla*_NDM-1_ and *bla*_SHV-12_ was isolated. It remains unclear where the exact origin of emergence of IncX3 plasmids carrying *bla*_NDM-1_ and *bla*_SHV-12_ is. More studies are warranted to characterise the prevalence of IncX3 plasmids in the *Enterobacteriaceae* and to understand the emergence and spread of IncX3 plasmids^23^.

**Figure 3:**
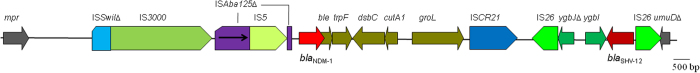
Genetic context of *bla*_NDM-1_ in *E. **c**oli* strain WCHEC13-8. IS*Aba125* was interrupted by the insertion of IS*5*. Genes shown from the left in order are *mpr* (encoding a zinc metalloproteinase), *bla*_NDM-1_, *ble* (mediating bleomycin resistance), *trpF* (encoding a phosphoribosylanthranilate isomerase), *dsbC* (encoding an oxidoreductase), *ctuA1* (encoding an ion tolerant protein), *groL* (encoding a chaperonin subunit), *ygbI* (a putative dehydrogenase gene), a truncated *ygbJ* (a putative DEOR-type transcriptional regulator gene), *bla*_SHV-12_ and a truncated *umuD* (encoding a mutagenesis protein).

The genetic context of *bla*_NDM-1_ on pNDM1_EC8 is identical to those on pNDM-HF727 and several other IncX3 plasmids from China. In such a context, *bla*_NDM-1_ is adjacent to an IS*5*-interrupted IS*3000*-truncated IS*Aba125* at upstream and is linked with *bla*_SHV-12_ at downstream (Fig. 3). *bla*_SHV-12_ is flanked by IS*26*, which could form a composite transposon with the potential to mobilise *bla*_SHV-12_ into different locations.

## Conclusions

An ST3835 *E. coli* clinical isolate carrying multiple genes, e.g. *bla*_NDM-1_, *bla*_CTX-M-15_, *bla*_CMY-42_ and *bla*_SHV-12_, which are able to confer resistance to broad-spectrum cephalosporins, was identified in China. Carbapenemase gene *bla*_NDM-1_ was carried by a self-transmissible IncX3 plasmid in this strain. IncX3 plasmids may have served as a common vehicle mediating the dissemination of *bla*_NDM_ among the *Enterobacteriaceae*. The recently-identified plasmid-born AmpC gene *bla*_CMY-42_ was in a complex genetic context, which was formed by the transposition of IS*Ecp1* and the insertion of two copies of IS*1* followed by homologous recombination between the two IS*1*.

## Material and Methods

### Strain and *in vitro* susceptibility

Strain WCHEC13-8 was a clinical isolate recovered in West China Hospital of Sichuan University, Chengdu, China. Species identification and *in vitro* susceptibility test were performed using the Vitek II automated system (bioMerieux, Lyon, France). MICs of imipenem, meropenem, amikacin, ceftazidime and ciprofloxacin were also determined using the microdilution broth method followed recommendations of the Clinical Laboratory Standards Institute^24^.

### Detection of carbapenemase-encoding genes

The strain was screened for acquired carbapenemase-encoding genes *bla*_GES_, *bla*_KPC_, *bla*_IMP_, *bla*_IMI_, *bla*_NDM_, *bla*_OXA-48_ and *bla*_VIM_ using PCR as described previously^25^,^26^,^27^,^28^. The complete coding sequence of *bla*_NDM_ was amplified with an additional pair of primers (NDM-up/NDM-dw)^25^ and the amplicon was sequenced in both directions using an ABI 3730xl DNA Analyzer (Applied Biosystems, Foster City, CA, USA) at the Beijing Genomics Institute (Beijing, China).

### Genome sequencing and analysis

Genomic DNA of strain WCHEC13-8 was prepared using QIAamp DNA Mini Kit (Qiagen, Hilden, Germany) and was subjected to whole genome sequencing with a ca. 140 × coverage using the Hiseq 2500 Sequencer (Illumina, San Diego, CA, USA) following the manufacturer’s protocol at the Beijing Genomics Institute. Reads were assembled to contigs using the Spades program^29^. The Prokka program^30^ was employed for annotating the genomic sequence. To identify whether this strain had unique genes, its assembled sequence was compared to the complete chromosome sequences of all of the 83 *E. coli* strains with their complete genome available in GenBank using the Gegenees program^31^. Potential unique genes were called using a stringent threshold of a 0.9 or higher score generated by Gegenees and then were confirmed or rejected using the BLAST program (http://blast.ncbi.nlm.nih.gov) against the 2,159 draft genomes of *E. coli* (accessed on December 18, 2014) to test whether they were absent from the *E. coli* draft genome database or not. Function of the products encoded by unique genes was predicted using both the Protein BLAST and InterProScan (http://www.ebi.ac.uk/interpro/) programs. The plasmids carrying *bla*_NDM-1_, *bla*_CMY-42_ or *bla*_CTX-M-15_ were completely circularised in sequence with intervals between contigs being filled by PCR with primers designed based on available sequences and Sanger sequencing.

### Strain typing

Phylogenetic group (A, B1, B2 and D) typing was performed as described previously^32^. Strain WCHEC13-8 was assigned to a ST using the assembled genomic sequence to query the seven alleles of the multi-locus sequence typing scheme for *E. coli* (http://mlst.warwick.ac.uk/mlst/dbs/Ecoli)^33^. Clonal complexes were assigned according to the founder ST identified using eBURST.

### Mating experiments and plasmid typing

Conjugation experiment was carried out in broth using azide-resistant *E. coli* strain J53 as the recipient. Transconjugants were selected on plates containing 4 μg/ml meropenem plus 150 μg/ml sodium azide. The presence of *bla*_NDM_ in transconjugants was confirmed using PCR and ERIC-PCR was used for further distinguishing transconjugants from the donor strain. Plasmid DNA that was prepared from the transconjugant using alkaline lysis was subjected to PCR-based replicon typing (PBRT)^34^. Sequences of IncX3 plasmids were retrieved from GenBank and aligned using MEGA (version 6). Maximum likelihood phylogenetic tree was constructed using MEGA using backbone sequences. IncI1 plasmid was assigned to ST and the replicon types of the IncF plasmids were assigned after querying their sequence against the plasmid MLST databases (http://pubmlst.org/plasmid/). Plasmids of the same type as pCTXM15_EC8 were identified using concatenated sequence of the IncF replicon alleles of pCTXM15_EC8 for BLAST.

### S1-PFGE

S1-PFGE was performed to determine the number and size of plasmids carried by strain WCHEC13-8 as described previously^35^. Briefly, agarose plugs containing whole-cell DNA of strain WCHEC13-8 were treated with 8 U of S1 nuclease (Fermentas, Thermo Scientific; Waltham, MA, US) and the reaction was stopped by adding 0.5 M EDTA (pH 8). PFGE was conducted with a 1% SeaKem Gold agarose gel (Lonza, Basal, Switzerland) using a CHEF DRII system (Bio-Rad, Hercules, CA, US) at 14°C, with a 6-V/cm current and run times of 12 h at switch time of 5 to 40 s followed by 8 h at switch time of 3 to 8 s. MidRange I PFG Marker (NEB, Ipswich, MA, US) was used for size estimation.

#### Nucleotide sequence accession number

Reads and the Whole Genome Shotgun project of WCHEC13-8 genomic sequence have been deposited into DDBJ/EMBL/GenBank under accession SRR2012643 and LCWG00000000, respectively. The sequence of pCMY42_EC8 and pCTXM15_EC8 has been deposited into DDBJ/EMBL/GenBank under accession numbers KP789019 and KP789020, respectively.

## Additional Information

**How to cite this article**: Feng, Y. *et al*. *Escherichia coli *of sequence type 3835 carrying *bla*_NDM-1_, *bla*_CTX-M-15_, _blaCMY-42_ and *bla*_SHV-12_. *Sci. Rep*. **5**, 12275; doi: 10.1038/srep12275 (2015).
